# Procoagulatory State in Inflammatory Bowel Diseases Is Promoted by Impaired Intestinal Barrier Function

**DOI:** 10.1155/2015/189341

**Published:** 2015-02-12

**Authors:** Luca Pastorelli, Elena Dozio, Laura Francesca Pisani, Massimo Boscolo-Anzoletti, Elena Vianello, Nadia Munizio, Luisa Spina, Gian Eugenio Tontini, Flora Peyvandi, Massimiliano Marco Corsi Romanelli, Maurizio Vecchi

**Affiliations:** ^1^Gastroenterology and Gastrointestinal Endoscopy Unit, IRCCS Policlinico San Donato, 20097 San Donato Milanese, Italy; ^2^Department of Biomedical Sciences for Health, University of Milan, 20131 Milan, Italy; ^3^Angelo Bianchi Bonomi Hemophilia and Thrombosis Centre, IRCCS Ca' Granda Ospedale Maggiore Policlinico, 20122 Milan, Italy; ^4^Department of Medical and Surgical Pathophysiology and Transplantations, University of Milan, 20122 Milan, Italy; ^5^Operative Unit of Laboratory Medicine, IRCCS Policlinico San Donato, 20097 San Donato Milanese, Italy

## Abstract

Inflammatory and immune mediated disorders are risk factors for arterial and venous thromboembolism. Inflammatory bowel diseases (IBD) confer an even greater risk of thromboembolic events than other inflammatory conditions. It has been shown that IBD patients display defective intestinal barrier functions. Thus, pathogen-associated molecular patterns (PAMPs) coming from the intestinal bacterial burden might reach systemic circulation and activate innate immunity receptors on endothelial cells and platelets, promoting a procoagulative state. Aim of the study was to test this hypothesis, correlating the presence of circulating PAMPs with the activation of innate immune system and the activation of the coagulatory cascade in IBD patients. Specifically, we studied lipopolysaccharide (LPS), Toll-like receptor (TLR) 2, TLR4, and markers of activated coagulation (i.e., D-Dimer and prothrombin fragment F1*+2*) in the serum and plasma of IBD patients. We found that LPS levels are increased in IBD and correlate with TLR4 concentrations; although a mild correlation between LPS and CRP levels was detected, clinical disease activity does not appear to influence circulating LPS. Instead, serum LPS correlates with both D-Dimer and F1*+2* measurements. Taken together, our data support the role of an impairment of intestinal barrier in triggering the activation of the coagulatory cascade in IBD.

## 1. Introduction

Cardiovascular events, including both arterial and venous thromboembolism, are a major cause of death and morbidity in Western countries [1]. Several cofactors influence the risk of cardiovascular events and, among these, inflammation seems to play a relevant role [2]. In fact, systemic and localized inflammatory processes can accelerate atherosclerosis and cause a hypercoagulative state [3]. As a matter of fact, the atherosclerotic plaque is ultimately an inflammatory lesion [4] where activated macrophages sustain the damage [5] and the inflammatory and coagulative molecular cascades are strictly linked and share several common mediators [3]. As such, it is not surprising that a wealth of epidemiologic data demonstrates an increased prevalence of thromboembolic complications in inflammatory and immune-mediated disorders [6]. Remarkably, it has been shown that, among many inflammatory and/or immune-mediated conditions, inflammatory bowel diseases (IBD), namely, ulcerative colitis (UC) and Crohn's disease (CD), confer the most prominent risk of thromboembolism [7].

IBD are chronic and relapsing inflammatory diseases of the gut and their exact etiology is still obscure [8]; however, several key events in the pathogenesis of IBD are well recognized and studied. It is commonly accepted that the onset of IBD is linked to the presence of an altered intestinal permeability [9], a reduced mucosal innate immunity function [9], and an exaggerated adaptive immune response [10].

The increased incidence of thromboembolic events in IBD patients is likely to be multifactorial and the inflammatory process may have itself a role together with vitamin deficiencies (i.e., vitamin B12, vitamin B6, and folate) and other acquired factors [11–13]. Moreover, a defective intestinal mucosa barrier function may as well contribute to the procoagulative state, specifically in IBD patients. In fact, a leaky epithelial layer and/or a defective innate immune response impair gut capability to control intestinal bacterial burden, allowing bacterial components to penetrate into the intestinal mucosa [9], reach the systemic circulation, and come into contact with endothelial cells and platelets. Those bacterial components, such as lipopolysaccharide (LPS), may work as pathogen-associated molecular patterns (PAMPs), that is, molecules associated with different groups of microorganisms, and after being recognized by cells they activate a prompt innate immune response [14]. In fact, immune and nonimmune cells present on their membrane specific receptors deputed to recognize PAMPs, such as Toll-like receptors (TLRs) [14]. Apart from membrane-bound TLRs, soluble TLRs were also described. These molecules can be found in body fluids upon bacterial sensing [15–17] and, acting as decoy receptors for PAMPs, they negatively regulate membrane-bound TLR activation in order to prevent exaggerated innate immune activation [18–20]. Interestingly, soluble TLRs have been proposed as potential biomarkers for several inflammatory/infectious conditions, including IBD [21–26].

Both endothelial cells and platelets possess innate immunity receptors, such as TLR2 and TLR4 [27–29], that, after being activated by PAMPs, promote cell activation and the subsequent release of different procoagulative molecules, all of them triggering the molecular pathways of coagulation.

In the present paper, we evaluated the potential link between decreased intestinal barrier function and activation of coagulation in IBD. To this aim we explored the correlation between circulating LPS, soluble TLR2 and TLR4 serum levels, and sensitive markers of activated coagulation (i.e., D-Dimer and prothrombin fragment F1 + 2) [30], thus evaluating the final steps of the coagulatory cascade.

## 2. Materials and Methods

### 2.1. Patients

After having read and signed a specific informed consent, 58 consecutive IBD patients (35 CD and 23 UC) followed up at the Gastroenterology and Gastrointestinal Endoscopy Unit of the IRCCS Policlinico San Donato and 20 healthy controls were enrolled in the study. Blood was collected from all IBD patients and all control subjects. All IBD diagnoses had been confirmed by standardized clinical, endoscopic, and histologic criteria [31, 32]. Disease activity was assessed using the Harvey-Bradshaw Index (HBI) for CD patients [33] and the Mayo scores for UC [34]. Patients with proctitis were excluded from the study. The demographic and clinical characteristics of the patients are reported in Table 1. The study was designed to respect the ethical guidelines of the Declaration of Helsinki. The Internal Review Board of the local Ethical Committee approved the study protocol (Ethical Committee Protocol number # 2025, ASL Milano-2, approved on June 14, 2007).

### 2.2. Blood Sampling and Serum and Plasma Collection

Peripheral blood was collected by venipuncture of an antecubital vein without any blood stasis using sterilized needles in BD vacutainer SST II Advance for serum collection and in BD vacutainer with sodium citrate for plasma collection. A 3 mL blood tube was used for serum and plasma separation after centrifugation at 2370 g for 15 min at room temperature. Serum and plasma were stored at −80°C in 0.5 mL aliquots until the time of assays.

### 2.3. C Reactive Protein Measurement

C reactive protein (CRP) levels were measured by a commercially available immunoturbidimetric assay (Roche Diagnostic, Germany).

### 2.4. TLR2 and TLR4 Protein Quantification in Serum

The measurement of TLR2 and TLR4 was performed by means of commercially available sandwich enzyme immunoassay (R&D System, USA, and USCN Life Sciences Inc., China, resp.), following manufacturers' instructions.

### 2.5. LPS Quantification

The Endpoint Chromogenic Limulus Amebocyte Lysate (LAL) Test (Lonza) was used as a quantitative test for LPS, according to manufacturer's instructions. Briefly, serum was diluted 1 : 10 with LAL reagent water (LRW), heat inactivated in a water bath for 15 minutes at 70°C, and then diluted 1 : 2 in LRW. The test was performed in a microplate at 37°C in heating block. Each sample was tested in duplicate. Fifty *μ*L of diluted sample was mixed with 50 *μ*L of the LAL supplied in the test kit and incubated at 37°C (±1°C) for 10 minutes. One hundred *μ*L of substrate solution was then mixed with the LAL sample and incubated at 37°C (±1°C) for an additional 6 minutes. The reaction was stopped with 100 *μ*L acetic acid 25% v/v in water. The absorbance of the sample was determined spectrophotometrically at 405–410 nm.

### 2.6. D-Dimer and F1 + 2 Dosage

D-Dimer HS kit (Instrumentation Laboratory, Spain) was used for the quantitative determination of D-Dimer in human citrated plasma, following manufacturer's instructions. Briefly, the D-dimer HS 500 latex reagent is a suspension of polystyrene latex particles of uniform size coated with the F(ab′)_2_ fragment of a monoclonal antibody highly specific for the D-Dimer domain included in fibrin soluble derivatives. Plasma containing D-Dimer was mixed with the latex reagent and the reaction buffer, obtaining agglutination directly proportional to the D-Dimer concentration.

ENzygnost F1 + 2 sandwich ELISA test (Siemens, Germany) was used to measure prothrombin fragment F1 + 2, according to manufacturer's instructions.

### 2.7. Statistical Analysis

Data were analyzed by use of a computerized program (GraphPad Prism, GraphPad Software Inc., San Diego, CA). Statistical methods employed included the use of Mann-Whitney Test and Spearman Correlation Test. Data are presented as the median and interquartile range. The statistical significance was set at *P* < 0.05.

## 3. Results

### 3.1. Circulating LPS Is Increased in IBD Patients and Correlates with TLR2 and TLR4 Serum Concentration in UC

**Figure 1:**
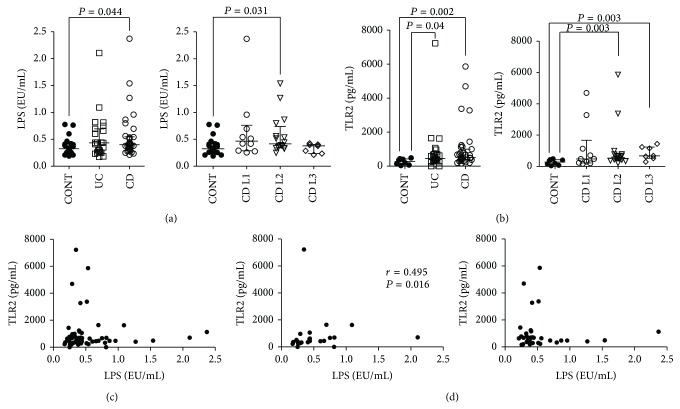
Circulating LPS and TLR2 are increased in IBD and correlate with each other in UC. Concentrations of LPS and TLR2 were measured in the sera of IBD (both UC and CD) patients and healthy controls (CONT). (a) LPS serum levels in CONT, UC, and CD patients (left panel) and CD locations according to Montreal classification (right panel). (b) TLR2 serum levels in CONT, UC, and CD patients (left panel) and CD locations according to Montreal classification (right panel). (c) Correlation between circulating levels of LPS and TLR2 in IBD patients. (d) Correlation between circulating levels of LPS and TLR2 in UC (left panel) and CD (right panel) patients. Horizontal bars in (a) and (b) represent median and interquartile range. Statistical analysis was performed by means of Mann-Whitney Test and Spearman Correlation Test. *P* < 0.05 was considered statistically significant.

**Figure 2:**
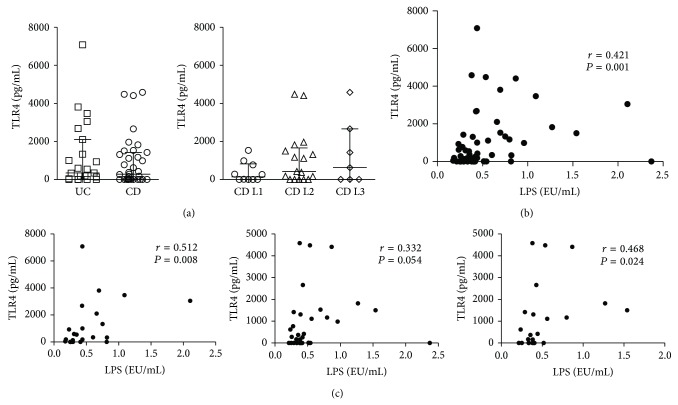
TLR4 serum correlates with circulating LPS in IBD patients. Concentrations of LPS and TLR4 were measured in the sera of IBD (both UC and CD) patients. (a) TLR4 serum levels in UC and CD patients (left panel) and CD locations according to Montreal classification (right panel). (b) Correlation between circulating levels of LPS and TLR4 in IBD patients. (c) Correlation between circulating levels of LPS and TLR4 in UC (left panel), CD (central panel) patients, and CD patients with colonic and ileocolonic involvement (L2 + L3 according to Montreal classification) (right panel). Horizontal bars in (a) represent median and interquartile range. Statistical analysis was performed by means of Spearman Correlation Test. *P* < 0.05 was considered statistically significant.

In order to evaluate the penetration of PAMPs in the blood flow, as a result of a defective intestinal barrier function in IBD, we measured circulating LPS in the sera of IBD patients versus control subjects. LPS levels were more elevated in CD (0.400 (95% CI 0.333–0.540) EU/mL) and UC (0.430 (95% CI 0.276–0.677) EU/mL) patients compared with controls (0.325 (95% CI 0.264–0.411) EU/mL; *P* = 0.044 and *P* = 0.205, resp.) (Figure 1(a)). When performing a subanalysis of CD patients according to their disease location, as described by the Montreal classification [35], we detected significantly increased LPS levels in patients with colonic disease (0.420 (95% CI 0.350–0.620) EU/mL) compared to controls (*P* = 0.031) (Figure 1(a)). Then, we measured serum concentration of TLR2, as putative aspecific marker of systemic innate immune activation. Similarly to LPS, we found more abundant TLR2 in CD (517.21 (95% CI 412.07–890.73) pg/mL) and UC (447.60 (95% CI 339.90–704.41) pg/mL) sera versus controls (281.15 (95% CI 122.23–412.05) pg/mL; *P* = 0.002 and *P* = 0.040, resp.) (Figure 1(b)). Interestingly, only CD patients with colonic or ileocolonic involvement showed increased TLR2 levels (512.80 (95% CI 436.92–778.27) and 656.72 (95% CI 578.43–1195.55) pg/mL, resp.; *P* = 0.003 and *P* = 0.003, resp.) compared to controls. We next analyzed the correlation between LPS and TLR2 levels; although we did not find any correlation between these two variables, when considering the whole IBD patients (Figure 1(c)) or CD patients (Figure 1(d)), a significant correlation (*r* = 0.495, *P* = 0.016) was observed when we considered UC patients separated from CD ones (Figure 1(d)). In addition, subanalysis of CD patients according to disease location did not reveal any further association (data not shown). We also evaluated the correlation between serum TLR4 (Figure 2(a)), that is, the principal innate immune receptor for LPS, and circulating LPS, and we detected a significant correlation (*r* = 0.421, *P* = 0.001) (Figure 2(b)); interestingly, when we repeated the analysis dividing patients according to their disease, we found more robust correlation for UC (*r* = 0.512, *P* = 0.008) and CD with colonic involvement (L2 + L3) (*r* = 0.468, *P* = 0.024) than for CD as a whole (*r* = 0.332, *P* = 0.054) (Figure 2(c)).

### 3.2. LPS Levels Correlate with Biochemical but Not Clinical Activity in IBD Patients, Whereas TLR2 and TLR4 Levels Are Independent of Disease Activity

**Figure 3:**
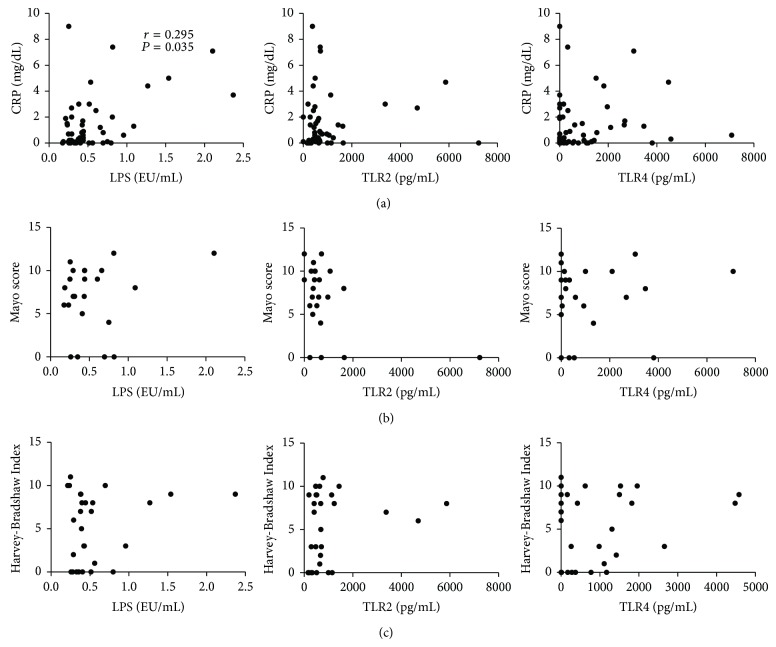
Circulating LPS correlates with biochemical but not clinical activity in IBD patients. Concentrations of LPS, TLR2, TLR4, and CRP were measured in the sera of IBD (both UC and CD) patients. Clinical activity of UC and CD was evaluated using Mayo score and Harvey-Bradshaw Index. (a) Correlation between CRP serum levels and circulating LPS, TLR2, and TLR4 in IBD patients. (b) Correlation between Mayo score and circulating LPS, TLR2, and TLR4 in UC patients. (c) Correlation between Harvey-Bradshaw Index and circulating LPS, TLR2, and TLR4 in CD patients. Statistical analysis was performed by means of Spearman Correlation Test. *P* < 0.05 was considered statistically significant.

In order to evaluate whether or not circulating LPS, TLR2, and TLR4 were merely markers of systemic inflammation or disease activity, we analyzed the correlation between those variables with a biochemical activity marker, that is, CRP, and two commonly used clinical activity indexes, such as the Mayo score for UC and the Harvey-Bradshaw Index for CD. Our analysis was able to demonstrate only a weak correlation between circulating LPS and CRP concentrations (*r* = 0.295, *P* = 0.035) (Figure 3). We observed no correlation at all when subanalysis for disease and disease location was performed (data not shown), suggesting that LPS, TLR2, and TLR4 levels are mostly independent of disease activity. No correlations have been observed with the clinical indexes (i.e., Mayo score and Harvey-Bradshaw Index) (Figure 3).

### 3.3. Concentrations of Circulating LPS Correlate with Plasma Levels of Markers of Activated Coagulation

**Figure 4:**
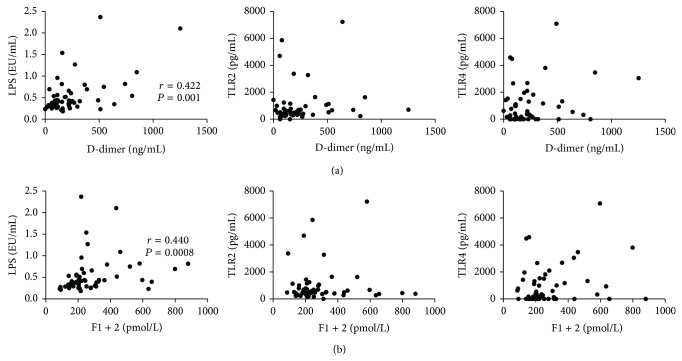
Circulating LPS correlates with markers of activated coagulation in IBD patients. Concentrations of LPS, TLR2, and TLR4 and levels of D-Dimer and prothrombin fragment F1 + 2 were measured in the sera and plasma, respectively, of IBD patients. (a) Correlation between D-Dimer plasma levels and serum LPS, TLR2, and TLR4 in IBD patients. (b) Correlation between prothrombin fragment F1 + 2 and circulating LPS, TLR2, and TLR4 in IBD patients. Statistical analysis was performed by means of Spearman Correlation Test. *P* < 0.05 was considered statistically significant.

**Figure 5:**
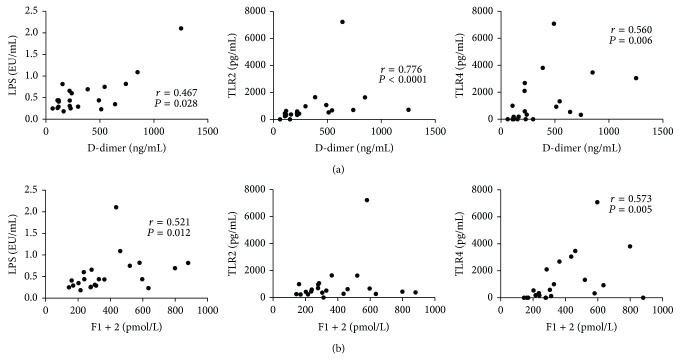
Circulating LPS, TLR2, and TLR4 correlate with markers of activated coagulation in UC patients. Concentrations of LPS, TLR2, and TLR4 and levels of D-Dimer and prothrombin fragment F1 + 2 were measured in the sera and plasma, respectively, of UC patients. (a) Correlation between D-Dimer plasma levels and serum LPS, TLR2, and TLR4 in UC patients. (b) Correlation between prothrombin fragment F1 + 2 and circulating LPS, TLR2, and TLR4 in UC patients. Statistical analysis was performed by means of Spearman Correlation Test. *P* < 0.05 was considered statistically significant.

**Figure 6:**
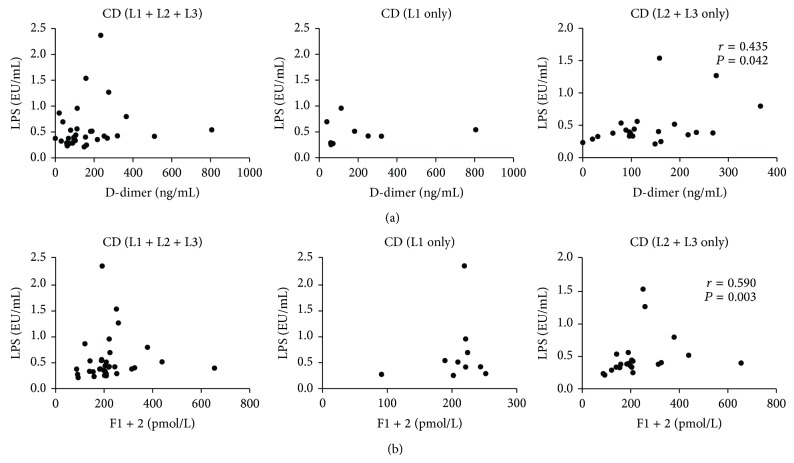
Circulating LPS correlates with markers of activated coagulation in CD patients with colonic involvement. Concentrations of LPS and levels of D-Dimer and prothrombin fragment F1 + 2 were measured in the sera and plasma, respectively, of CD patients. (a) Correlation between D-Dimer plasma levels and serum LPS in all CD patients (left panel), CD patients with exclusive ileal involvement (L1 according to Montreal classification) (central panel), and CD patients with colonic involvement (L2 + L3 according to Montreal classification). (b) Correlation between prothrombin fragment F1 + 2 and circulating LPS in all CD patients (left panel), CD patients with exclusive ileal involvement (L1 according to Montreal classification) (central panel), and CD patients with colonic involvement (L2 + L3 according to Montreal classification). Statistical analysis was performed by means of Spearman Correlation Test. *P* < 0.05 was considered statistically significant.

We also measured the plasma concentrations of D-Dimer and prothrombin fragment F1 + 2, which are well known markers of activated coagulation and are increased in procoagulative states. LPS levels significantly correlated with both D-Dimer (*r* = 0.422, *P* = 0.001) and F1 + 2 concentrations (*r* = 0.440, *P* = 0.0008) (Figure 4); however, no correlation was found between TLR2 or TLR4 and those coagulation markers (Figure 4). On the other hand, when we analyzed only data obtained in UC patients, we not only confirmed the correlation between LPS and both D-Dimer (*r* = 0.467, *P* = 0.028) and F1 + 2 (*r* = 0.521, *P* = 0.012), but also detected good correlations between TLR2 and D-Dimer (*r* = 0.776, *P* < 0.0001), TLR4 and D-Dimer (*r* = 0.560, *P* = 0.006), and TLR4 and F1 + 2 (*r* = 0.573, *P* = 0.005) (Figure 5). When all CD patients were considered for analysis, no significant correlation was found; remarkably, when only patients with colonic disease (L2 and L3) were taken into account, correlations between D-Dimer and F1 + 2 and LPS levels were confirmed (*r* = 0.435, *P* = 0.042, and *r* = 0.590, *P* = 0.003, resp.) (Figure 6).

## 4. Discussion

Since 1972, when Shorter et al. postulated that a primary defect in gut permeability and barrier function may lead to the onset of persistent inflammation in the gut and to the development of IBD [36], a growing body of evidence has demonstrated that the impairment of intestinal barrier function is a common feature in IBD patients leading to derangements in both epithelial permeability to gut antigens and early mucosal innate immune responses, protecting from microorganisms penetration and bacterial translocation [8, 9]. Interestingly, genetic studies demonstrated a strong correlation between the carriage of polymorphisms of genes regulating intestinal epithelial paracellular permeability and the risk of developing UC [37, 38], whereas being carrier of innate immunity-related gene polymorphisms increases the susceptibility to CD [38].

Patients suffering from IBD, and particularly UC [39], are more prone to incur into thromboembolic events [40], similarly to patients affected by other inflammatory and immune-related disorders [6]; however, the only presence of an ongoing inflammatory process does not explain by itself this phenomenon. In fact, it has been shown that the risk of thromboembolism is more elevated in IBD than rheumatoid arthritis, a disease often characterized by a prominent systemic inflammation [7], thus suggesting that some gut specific mechanisms may be involved. Besides deficiencies of vitamins involved in the proper regulation of coagulatory homeostasis, such as vitamin B6, vitamin B12, and folic acid [11–13], the alteration of intestinal barrier function may be a cofactor promoting a procoagulative state in IBD.

The data presented in this paper strongly support this novel hypothesis. In fact, in order to assess intestinal permeability to PAMPs and bacterial translocation from the gut, we measured circulating LPS and found higher concentrations of this bacterial component in the sera of IBD patients (Figure 1(a)). Consistent with the data previously presented by Candia et al. [26], IBD patients also presented higher serum levels of TLR2, as a sign of innate immune activation (Figures 1(b), 1(c), and 1(d)). Remarkably, serum TLR2 was recently described to correlate with the presence of prosthetic joint infection in patients undergoing revision of joint arthroplasty, because of suspected local infection [23]; thus, circulating TLR2 may be considered a marker of the presence of bacterial components in the blood flow. Moreover, high levels of soluble TLR2 were shown in the sera of patients affected by psoriasis [21] and systemic lupus erythematosus [25], diseases in which innate immunity plays a major pathogenetic role [41, 42]. As such, high levels of soluble TLR2 may reflect the activation of innate inflammatory responses.

LPS levels correlated with the serum concentrations of TLR4, which is the innate immune receptor deputed to LPS recognition (Figure 2). This may be a sign of an increased expression and activation of TLR4, because of LPS binding.

Apart from a very modest correlation between LPS and CRP levels, LPS, TLR2, and TLR4 concentrations did not appear to be influenced by biochemical and clinical disease activity (Figure 3), suggesting that their levels may be influenced, for the great part, by intestinal permeability.

Both TLR2 and TLR4 are expressed by platelets and endothelial cells [27–29]; moreover the binding of their respective ligands causes the procoagulatory activation of these cell populations. More in detail, TLR2 signaling in platelets leads to a thromboinflammatory response, through the activation of phosphoinositide 3-kinase [43], cyclooxygenase, and purinergic P2Y1 and P2Y12 receptors [44] and alpha-granule release [45], whereas LPS-TLR4 binding enhances classical agonist-induced platelet aggregation [46, 47]. The TLR2 and TLR4 signaling on endothelial cells strongly activates NF-*κ*B [5, 29], leading to the release of proinflammatory mediators, which can activate the coagulatory cascade. Nonetheless, the pathogenesis of atherosclerotic plaques is mediated by the presence of activated macrophages within the plaque, which also respond vigorously to TLR stimulation releasing proinflammatory cytokines [48].

Given the multiplicity of cell types and pathways involved in the activation of coagulation mediated by TLRs, we decided to measure the activation products of the coagulation and fibrinolysis, that is, the fibrin degradation product D-Dimer and prothrombin fragment F1 + 2, as they describe closely the overall activation of the coagulatory cascade [30]. Remarkably, LPS levels significantly correlated with both D-Dimer and F1 + 2 (Figure 4), clearly suggesting that circulating PAMPs may function as triggers for the activation of coagulation.

It may be worthy to note that the correlation between circulating LPS and markers of activated coagulation was stronger when considering only UC patients (Figures 1(d), 2(c), and 5) and CD patients with colonic or ileocolonic disease (L2 and L3 according to Montreal classification [35]) (Figure 6). These data suggest that the presence of colonic disease is the key risk factor for coagulatory unbalance that may follow bacterial translocation from the gut; indeed, the colon has to cope with a significantly greater bacterial burden than the rest of the gastrointestinal tract; as such it is rational that a break-down of intestinal barrier function in this site might lead to a more prominent bacterial penetration/translocation.

Moreover, in order to explain the differences we found in UC versus CD, other issues should be taken into account: CD patients often display greater signs of systemic inflammation rather than UC; as such, data coming from CD patients may suffer from the noise resulting from inflammation-driven activation of coagulation; as an alternative, PAMPs-mediated coagulatory activation may have a different relevance or follow different pathways in UC versus CD, also considering that genetic data suggest different defects in intestinal barrier function in those two diseases [38].

Taken together, our data support the novel hypothesis that intestinal barrier defects contribute to the development of a procoagulatory state in IBD. Our results need to be confirmed with future studies; in fact, the correlations we observed in human patients do not demonstrate the biological importance of the phenomenon we are postulating. A further limitation of our study is that we are using very simple markers in order to explore extremely complex systems. Indeed, experiments on animal models of intestinal inflammation are warranted in order to provide the final demonstration of our hypothesis and mechanistically dissect it and evaluate its real biologic relevance.

## Figures and Tables

**Table 1:** Demographic and clinical characteristics of enrolled IBD patients.

Diagnosis	CD	UC	Controls
# of patients	35	23	20
Age (mean ± SD)	40.79 ± 16.68	45.39 ± 15.14	39.79 ± 13.24
Sex (male/female)	16/19	15/8	12/8
Location CD			
Ileal (L1)	11	—	—
Colonic (L2)	17	—	—
Ileocolonic (L3)	7	—	—
Upper GI (L4)	0	—	—
Extent UC			
Left sided colitis	—	6	—
Extensive colitis	—	17	—
Disease behavior CD			
Nonstricturing, nonpenetrating (B1)	21	—	—
Stricturing (B2)	8	—	—
Penetrating (B3)	1	—	—
Perianal disease	5	—	—
Previous resective surgery	2	—	—
Disease activity indexes			
Harvey-Bradshaw Index (mean ± SD)	5.37 ± 4.02	—	—
Mayo score (mean ± SD)	—	6.96 ± 3.85	—
Folate serum levels (mcg/L)	5.98 ± 2.02	6.56 ± 3.28	
Vitamin B12 serum levels (ng/L)	398.4 ± 230.0	330.8 ± 124.5	
